# Brain language networks and cognitive outcomes in children with frontotemporal lobe epilepsy

**DOI:** 10.3389/fnhum.2023.1253529

**Published:** 2023-10-27

**Authors:** Alejandra M. Hüsser, Phetsamone Vannasing, Julie Tremblay, Bradley Osterman, Anne Lortie, Paola Diadori, Philippe Major, Elsa Rossignol, Kassandra Roger, Solène Fourdain, Sarah Provost, Yara Maalouf, Dang Khoa Nguyen, Anne Gallagher

**Affiliations:** ^1^Neurodevelopmental Optical Imaging Laboratory (LIONlab), Research Center, Sainte-Justine Mother and Child University Hospital Center, Montreal, QC, Canada; ^2^Department of Psychology, Université de Montréal, Montreal, QC, Canada; ^3^Division of Neurology, Sainte-Justine Mother and Child University Hospital Center, Montreal, QC, Canada; ^4^Division of Pediatric Neurology, Montreal Children’s Hospital, McGill University Health Centre, Montreal, QC, Canada; ^5^Department of Neuroscience, Université de Montréal, Montreal, QC, Canada; ^6^Department of Pediatrics, Université de Montréal, Montreal, QC, Canada; ^7^CHUM Research Center, Université de Montréal, Montreal, QC, Canada

**Keywords:** language networks, receptive language, functional near-infrared spectroscopy (fNIRS), functional connectivity, electroencephalography (EEG), pediatric frontotemporal lobe epilepsy, neurodevelopment

## Abstract

**Introduction:**

Pediatric frontal and temporal lobe epilepsies (FLE, TLE) have been associated with language impairments and structural and functional brain alterations. However, there is no clear consensus regarding the specific patterns of cerebral reorganization of language networks in these patients. The current study aims at characterizing the cerebral language networks in children with FLE or TLE, and the association between brain network characteristics and cognitive abilities.

**Methods:**

Twenty (20) children with FLE or TLE aged between 6 and 18 years and 29 age- and sex-matched healthy controls underwent a neuropsychological evaluation and a simultaneous functional near-infrared spectroscopy and electroencephalography (fNIRS-EEG) recording at rest and during a receptive language task. EEG was used to identify potential subclinical seizures in patients. We removed these time intervals from the fNIRS signal to investigate language brain networks and not epileptogenic networks. Functional connectivity matrices on fNIRS oxy-hemoglobin concentration changes were computed using cross-correlations between all channels.

**Results and discussion:**

Group comparisons of residual matrices (=individual task-based matrix minus individual resting-state matrix) revealed significantly reduced connectivity within the left and between hemispheres, increased connectivity within the right hemisphere and higher right hemispheric local efficiency for the epilepsy group compared to the control group. The epilepsy group had significantly lower cognitive performance in all domains compared to their healthy peers. Epilepsy patients’ local network efficiency in the left hemisphere was negatively associated with the estimated IQ (*p* = 0.014), suggesting that brain reorganization in response to FLE and TLE does not allow for an optimal cognitive development.

## 1. Introduction

Focal epilepsy of the frontal (FLE) or temporal lobe (TLE) is the most frequent diagnosis in pediatric epilepsy (Hermann and Seidenberg, 2002; Gallagher and Lassonde, 2005; Beleza and Pinho, 2011; Téllez-Zenteno and Hernández-Ronquillo, 2012; Behr et al., 2016). Pediatric FLE and TLE have been shown to interfere with brain development and can result in altered functional cerebral networks including reorganization of brain circuits that support language functions (Berl et al., 2005, 2014; Mbwana et al., 2009; Hamberger and Cole, 2011; Gallagher et al., 2013). For instance, a more frequent bilateral hemispheric speech representation or right-hemispheric language dominance have been found in these children (Gallagher et al., 2008b, 2016; Baciu and Perrone-Bertolotti, 2015; Foley et al., 2020). Moreover, there is evidence of intra-hemispheric reorganization in patients with left FLE or TLE, where regions within the left hemisphere typically not involved in language production (e.g., posterior areas) are recruited during an expressive language task (Gallagher et al., 2007; Vannasing et al., 2016). Such alterations in children with FLE and TLE may reflect either language network reorganization allowed by early brain plasticity (Staudt et al., 2001, 2002; Hamberger and Cole, 2011) or aberrant development of language brain networks due to early neuropathology (Hermann et al., 2002; Vannest et al., 2009; Sepeta et al., 2015; Bear et al., 2019).

Major advances in neuroimaging data acquisition and functional connectivity (FC) analysis have allowed a more thorough investigation of brain network reorganization (Friston, 2011). Children (Sepeta et al., 2015; Chou et al., 2018; Vannest et al., 2019; Foley et al., 2020) and adults (Balter et al., 2019) with FLE or TLE show different FC patterns within their cerebral language networks compared to healthy controls. Precisely, reduced FC between homologous inferior (IFG) and middle (MFG) frontal gyri and posterior superior temporal gyri (PSTG) as well as decreased intra-hemispheric FC between the IFG and MFG in both hemispheres has been reported in children with left hemispheric focal epilepsy during a semantic congruency task compared to typically developing controls (Sepeta et al., 2015). In children with TLE specifically, FC within the left IFG during a story listening task was significantly lower than in healthy peers (Vannest et al., 2019). The strength of FC among language-related brain areas might therefore be an index of altered brain network in children with FLE or TLE.

The graph theory approach has gained increasing recognition as a mean to characterize brain networks in patients with epilepsy of all ages (Bernhardt et al., 2015; Farahani et al., 2019; Tavakol et al., 2019; Rodríguez-Cruces et al., 2020; Tung et al., 2021; Slinger et al., 2022). Graph theory allows the rederivation of common network properties from the FC matrices. Notably, it provides the network’s segregation and integration properties (Rubinov and Sporns, 2010), which refer to a network’s tendency to have locally specialized subnetworks (segregation), and to the efficacy of a network to efficiently globally integrate information from distinct parts of the network (integration; Rubinov and Sporns, 2010). A recent meta-analysis addressed the question of network alterations in pediatric and adult patients with focal epilepsy (Slinger et al., 2022). The authors included the results of 45 studies (*n* > 1,400) published between 2006 and 2020 that used a graph theoretical approach. Although the structural integration of neural networks was lower in patients with epilepsy than healthy controls, the network integration and segregation characteristics did not statistically differ between groups. The authors hypothesized that the high heterogeneity of individual results notably due to methodological or technological differences probably precluded the identification of network differences between groups. To reduce this heterogeneity, they performed sub-analyses that included only recent studies (2013 and after) and found a significantly lower network segregation for the epilepsy group compared to healthy controls. This difference was even more striking when comparing patients with TLE only, i.e., the largest subgroup of patients, to healthy controls. Lower network segregation suggests reduced local processing efficiency (Rubinov and Sporns, 2010), which may be a specific characteristic of cerebral networks in epilepsy patients.

In addition to altered brain networks, pediatric FLE and TLE have been associated with many behavioral, cognitive, and psycho-affective deficits impacting the well-being and quality of life of these children and their families (Hernandez et al., 2002; Berg et al., 2008; Wilson et al., 2015; Smith, 2016; Goodwin et al., 2017; Karrasch et al., 2017; Law et al., 2018). Although most children with FLE and TLE have intellectual abilities within the norms, some children have an overall decrease of their intellectual profile (Gallagher and Lassonde, 2005). Also, children with FLE and TLE often present deficits in specific cognitive domains including attention, executive functions, memory, language, and social cognition (Helmstaedter, 2001; Hermann and Seidenberg, 2002; Hernandez et al., 2002, 2003; Jokeit and Schacher, 2004; Gallagher and Lassonde, 2005; Prévost et al., 2006; Fuentes and Smith, 2015; Reuner et al., 2016). Although some cognitive difficulties are more specific to FLE (e.g., impaired executive functions) or TLE (e.g., memory problems), the neuropsychological profiles of these children are often heterogeneous and complexly intertwined (Smith, 2016; Law et al., 2018; Hermann et al., 2021). Common and frequent difficulties in children with FLE and TLE are language impairments affecting expressive (e.g., phonetic or semantic verbal fluency, naming) or receptive (e.g., phonological awareness, comprehension) language functions, or verbal problem-solving skills (Metternich et al., 2014; Teixeira and Santos, 2018; Bear et al., 2019). This is not surprising since language processing in healthy humans is thought to be organized in a complex cerebral network predominantly in the left hemisphere including posterior inferior frontal regions, large parts of the temporal lobe, the angular and supramarginal gyri in the parietal lobe, and white matter fiber pathways connecting frontotemporoparietal and occipitotemporal regions (Tremblay and Dick, 2016; Hickok et al., 2021; Hickok, 2022).

Typical development of language brain networks is a long process that starts *in utero* and evolves throughout infancy, childhood and even into late adolescence (Paquette et al., 2013, 2015; Weiss-Croft and Baldeweg, 2015; Skeide and Friederici, 2016). Overall, brain network topology in typically developing children is characterized by a decrease of network segregation with age, while global integration increases (Supekar et al., 2009; Gao et al., 2017). The establishment of language-related neuronal networks is accompanied by the development of language abilities that are crucial for later social, academic, and daily functioning. In children with focal epilepsies, the strength of left intra-hemispheric FC between the IFG and PSTG during a language task has been positively associated with language skills (Sepeta et al., 2015). This suggests that reductions of FC within regions of the brain’s language network might represent suboptimal cerebral reorganization associated with language impairments. Furthermore, graph theoretical studies in children and adolescents with focal epilepsies have shown that higher resting-state network segregation and integration are associated with higher general intellectual capacities (Paldino et al., 2017; Songjiang et al., 2021). A positive association between segregation and cognitive capacities differs from the typical developmental trajectory of network topology reported in healthy children (Giedd et al., 1999; Supekar et al., 2009). However, whether a similar atypical relationship exists between brain language network characteristics and language and cognitive functions in children with FLE or TLE specifically remains unknown.

Despite numerous studies reporting cerebral alterations in pediatric focal epilepsies, specific patterns of brain network reorganization are still unpredictable and the precise impact on cerebral language networks is not clear. Also, there is no consensus yet on the functional impact of specific language network alterations. The current study aims at characterizing the cerebral networks related to language processing in children with FLE or TLE as compared to healthy controls, using functional near-infrared spectroscopy (fNIRS) and a graph theoretical approach. Qualitative comparisons of fNIRS FC patterns between epilepsy subgroups (FLE vs. TLE, left vs. right epilepsy) and graph theory characteristics will also be explored. fNIRS has been widely used to investigate cerebral language processing because it allows to capture hemodynamic changes in cortical regions. It is particularly adapted for pediatric patients because it is non-invasive, relatively tolerant to movements, and offers the flexibility to acquire data when the caregiver remains close by. The secondary aim of this study is to characterize the association between language brain network characteristics and the neuropsychological profile of children with FLE and TLE.

This is the first study to explicitly focus on pediatric FLE and TLE, the two most common types of focal epilepsies. Based on the overlap between the epileptogenic network of these patients and the typical organization of cerebral language processing, children with FLE and TLE have a higher risk of alterations in brain language networks. Our findings will strengthen the current knowledge on FC alterations of brain language networks in these patients. Moreover, graph network analysis allows to uncover how alterations of FC translate to characteristics of graph network architecture. To our knowledge this is the first study that applies graph theory to characterize functional brain network topology in children with FLE and TLE. Moreover, this study will contribute to a better understanding of the cerebral alterations and cognitive abilities in children with FLE and TLE, i.e., the specific brain-behavior relationship under neuropathologic conditions.

## 2. Materials and methods

### 2.1. Participants

Between 2017 and 2020, we recruited 20 children with FLE (*n* = 9) or TLE (*n* = 11) and 29 healthy controls. All participants were French speakers, aged between 6 and 18 years, born at term, and had no history of previous neurosurgery, metabolic disorder, traumatic brain injury or intellectual disability. Medical files of patients with epilepsy followed at the Neurology Division of the Mother and Child University Hospital Center Sainte-Justine (CHUSJ, Montreal, QC, Canada) were screened. For children with epilepsy, exclusion criteria were: multifocal epilepsy, significant structural abnormalities with mass effect, genetic syndrome known to significantly affect neurodevelopment, autism spectrum disorder, multiple ( ≥ 3) comorbidities (e.g., attention deficit hyperactivity disorder, dyslexia, dyspraxia, etc.). Patients and their caregivers were approached by their neurologist (BO, AL, PD, PM, ER) and subsequently contacted by the research coordinator (AMH). The study’s protocol (2014-664, 3,876) was approved by the CHUSJ’s research ethics board. All parents and children provided written informed consent. Descriptive statistics of the sample’s sociodemographic and clinical characteristics are presented in Table 1.

**Table 1:** Descriptive statistics of the socio-demographic and clinical characteristics of participants who completed the neuropsychological assessment, the resting state and the passive story listening task during the fNIRS-EEG recording.

	Epilepsy patients	Healthy controls	Group comparison (value of *p*)^*^
	(Number of missing data)		
*N*	13	26	
Sex [female/male]	4/9	11/15	0.728
Age [M ± SD]	11.2 ± 3.9	12.9 ± 4.1	0.203
**Socio-economic status [Median]**			
Family income	5/5	5/5 (3)	0.793
Parental education	5/6	5.8/6	0.311
**Socio-affective well-being**^**a**^ **[M ± SD]**			
Self-concept	50.2 ± 7.1 (1)	52.1 ± 6.0	0.439
Symptoms of depression	49.8 ± 8.2 (1)	47.5 ± 5.5	0.343
Symptoms of anxiety	53.7 ± 8.5 (1)	48.1 ± 7.0	.056^1^
Handedness [right/left/ambidexter]	9/3/1	21/1/4	-
**Epilepsy diagnosis**			
Type [FLE or TLE]	5/8^b^	–	–
Lateralization [left/right/bilateral]	3/6/3^c^	–	–
Age of epilepsy onset [M ± SD]	6.4 ± 3.1 (1) Median = 5.7	–	–
Epilepsy duration [M ± SD]	4.8 ± 3.1 (1) Median = 4.1	–	–
Number of ASM [M ± SD]	1.3 ± 0.48	–	–
Seizure control [yes/no]	9/4	–	–

^*^Critical alpha: *p* = 0.05, ^1^statistical tendency (0.05 < *p* < 0.08). ^a^Measured with the Beck Youth Inventory (BYI-II) who was only completed by participants aged ≥7 years, *n^epi/ctrl^* = 10/23, ^b^Left/right/bilateral FLE: 1/3/1, left/right/bilateral TLE: 3/3/2, FLE, frontal lobe epilepsy; TLE, temporal lobe epilepsy; ASM, anti-seizure medication.

### 2.2. Procedure

Participation in the study included (1) a combined functional near-infrared spectroscopy (fNIRS) and electroencephalography (EEG) data acquisition at rest and during a receptive language task, and (2) a neuropsychological assessment. Both are described in the following sections.

#### 2.2.1. fNIRS-EEG data acquisition

**Figure 1:**
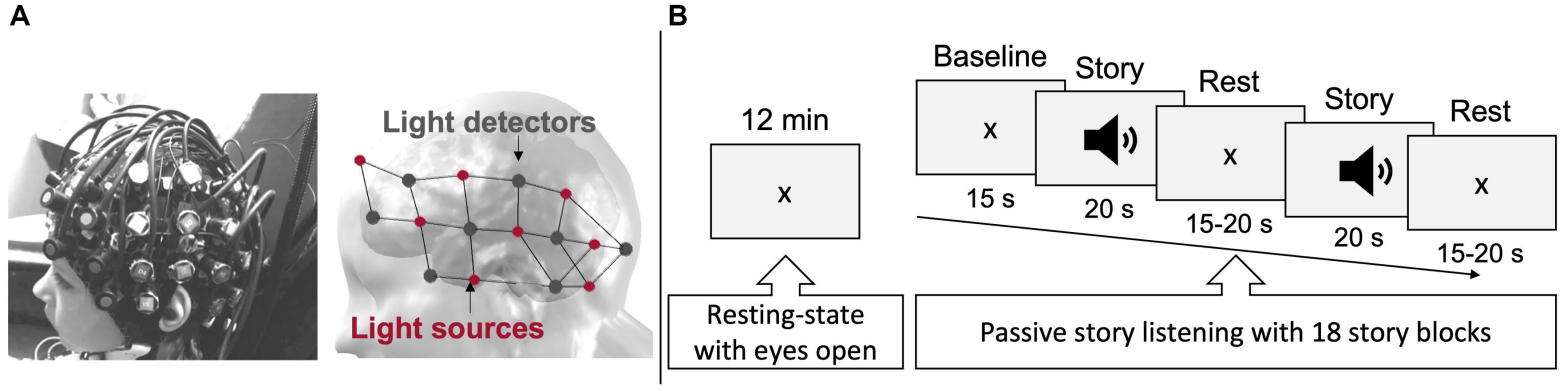
fNIRS-EEG recording set-up. **(A)** fNIRS-EEG montage as it was placed on a participant’s head (left), and the arrangement of 16 emitting light sources ( λ 1 | 2 = 760 | 850 nm, red dots) and 16 light detectors (dark grey dots) covering regions of interest over both hemispheres (right). This resulted in 50 measurement channels. **(B)** The two behavioral paradigms, i.e., 12-min resting-state period and the 12-min receptive language task, performed during combined fNIRS-EEG data collection.

The fNIRS-EEG data acquisition was performed using a continuous-wave fNIRS system (NIRScout, NIRx Medical Technologies, LLC, Berlin, Germany) equipped with 16 detectors and 16 sources emitting light at two-different wavelengths (λ1 | 2 = 760 | 850 nm), yielding to a total of 50 channels per wavelengths. The hemodynamic signal was recorded at a sampling rate of 7.8 Hz. Detectors and sources were held in place on an elastic cap (NIRScap, EASYCAP GmbH, Woerthsee-Etterschlag, Germany) and arranged according to the 10–20 system to cover frontal, temporal, and part of the parietal and occipital areas bilaterally (see Figure 1A). Spatial localization of all probes was digitalized on a head model using fiducial points (nasion, inion, LPA and RPA) as reference landmarks (Polaris Stereotaxic System, Northern Digital Inc., Waterloo, ON, Canada; Brainsight Frameless 39 software, Rogue Research Inc., Montreal, QC, Canada). Digitalized coordinates were projected onto a structural MRI template (Kazemi et al., 2007) and visualized with the 3DMTG interface from the LIONirs toolbox (Tremblay et al., 2022), allowing to project individual fNIRS data into a 3D MRI space.

Simultaneously to fNIRS data acquisition, EEG signal was acquired to identify potential subclinical seizures in patients, remove these time intervals from the fNIRS signal, and ensure we investigate language brain networks and not epileptogenic networks. EEG recording (BrainVision Recorder, Version 1.2, Brain Products GmbH, Gilching, Germany) included 21 active electrodes placed between the fNIRS fibers and arranged according to the 10–20 system, with FCz as the reference (actiCHamp system, Brain Products GmbH, Gilching, Germany). The electrophysiological signal was acquired at a sampling rate of 500 Hz.

The fNIRS-EEG recording took place in a dimmed-soundproof room. Participants sat in a comfortable armchair and were instructed to relax, avoid any intentional movements or muscular tension, and keep their gaze on the fixation cross presented in the center of a screen (placed at 114 cm in front of them). Throughout the entire session, a member of the research team sat next to the participant to give instructions and ensure that the participant remained engaged. Meanwhile the quality of the fNIRS-EEG signal and the state of alertness of the participant were monitored by a senior NIRS-EEG technician (PV). fNIRS-EEG data was acquired during two conditions (Figure 1B): (1) a consecutive 12-min resting-state with eyes open, and (2) a 12-min passive story listening task previously validated for receptive language-related brain activation (Bassett et al., 2008; Gallagher et al., 2008a, 2012, 2016; Vannasing et al., 2016). This paradigm had the advantage to minimize movements, minimizing signal artifacts and the need for signal preprocessing and modifications. The story was an abbreviated version of Snow White narrated in French and presented throughout 18 individual segments in a block design paradigm, where periods of rest and task alternated (Presentations®, Neurobehavioral Systems, 2018, Berkeley, CA, United States). The interstimulus interval was pseudo randomized between 15 and 20 s and stimulus presentation was ~20 s. After the fNIRS-EEG recording, participants responded to 12 questions verifying if they followed the storyline. A video recording of the fNIRS-EEG session enabled visual support during offline pre-processing for the identification of movements and potential epileptic seizures.

#### 2.2.2. Neuropsychological assessment

All participants underwent a neuropsychological evaluation that included an estimation of the general intellectual functioning (estimated IQ) and the assessment of the receptive and expressive language abilities. As proposed in the Wechsler Abbreviated Scale of Intelligence (WAIS-II; Wechsler, 2011), the estimated-IQ was computed based on four subtests of the Wechsler intelligence scale for children (6–16 years, WISC-IV; Wechsler, 2005) and adults (16 years and over, WAIS-IV; Wechsler, 2010): Vocabulary (word knowledge), Similarities (verbal reasoning), Block design (visuo-constructive abilities) and Matrix reasoning (non-verbal problem solving). The receptive and expressive language abilities were assessed using the Peabody Picture Vocabulary Test, revised version (PPVT-R; Dunn et al., 1993) and the One-Word Picture Vocabulary Test (EOWPVT-2000; Brownell, 2000), respectively. All these tests are standardized validated tools frequently used for neuropsychological assessment of children with epilepsy. Individual test results were transformed into T scores based on the norms for the French-Canadian population.

In addition, three questionnaires were completed. First, a homemade demographic, developmental and socio-economic inventory was completed by the primary caregiver prior to the first meeting to confirm the eligibility of the child and determine the family socio-economic status (SES). The family’s income (scaled from 1 to 5) and the parental education as defined by the mean of the maternal and paternal level of education (scaled from 1 to 6) served as indices for the SES (American Psychological Association, 2022). Second, the assessor completed the Beck Youth Inventory (BYI-II; Beck et al., 2005) with the child (aged 7 and over) to assess three factors of the participants’ psychological-wellbeing: self-concept, depression, and anxiety. Since impaired self-concept and psychological problems have been shown to be more prevalent in the epileptic population (Reilly et al., 2014; Puka et al., 2020) and can have an impact on the cognitive profile (Rock et al., 2018), these scores allowed to detect symptoms of psychological distress and control for differences between study groups if needed. Third, a French version of the Edinburgh Handedness Inventory (Oldfield, 1971) was also completed by the child to determine each participant’s handedness based on self-reported daily activities. Left-handedness and ambidexterity have been associated with an increased incidence of atypical hemispheric representation of language functions (Knecht et al., 2000).

### 2.3. fNIRS-EEG data preprocessing

The raw EEG signal was filtered (bandpass range: 0.3–35 Hz, notch: 60 Hz) and screened for potential neurophysiological abnormalities by a clinical epileptologist (BO) (BrainVision Analyzer, Version 2.2, Brain Products GmbH, Gilching, Germany). This aimed to identify sub-clinical seizures in the participants with epilepsy and assure the absence of pathological findings within the control group. fNIRS data recorded simultaneously to a subclinical seizure identified on the EEG were excluded from further analyses to avoid contamination of the epileptogenic networks when mapping the language brain networks.

**Figure 2:**
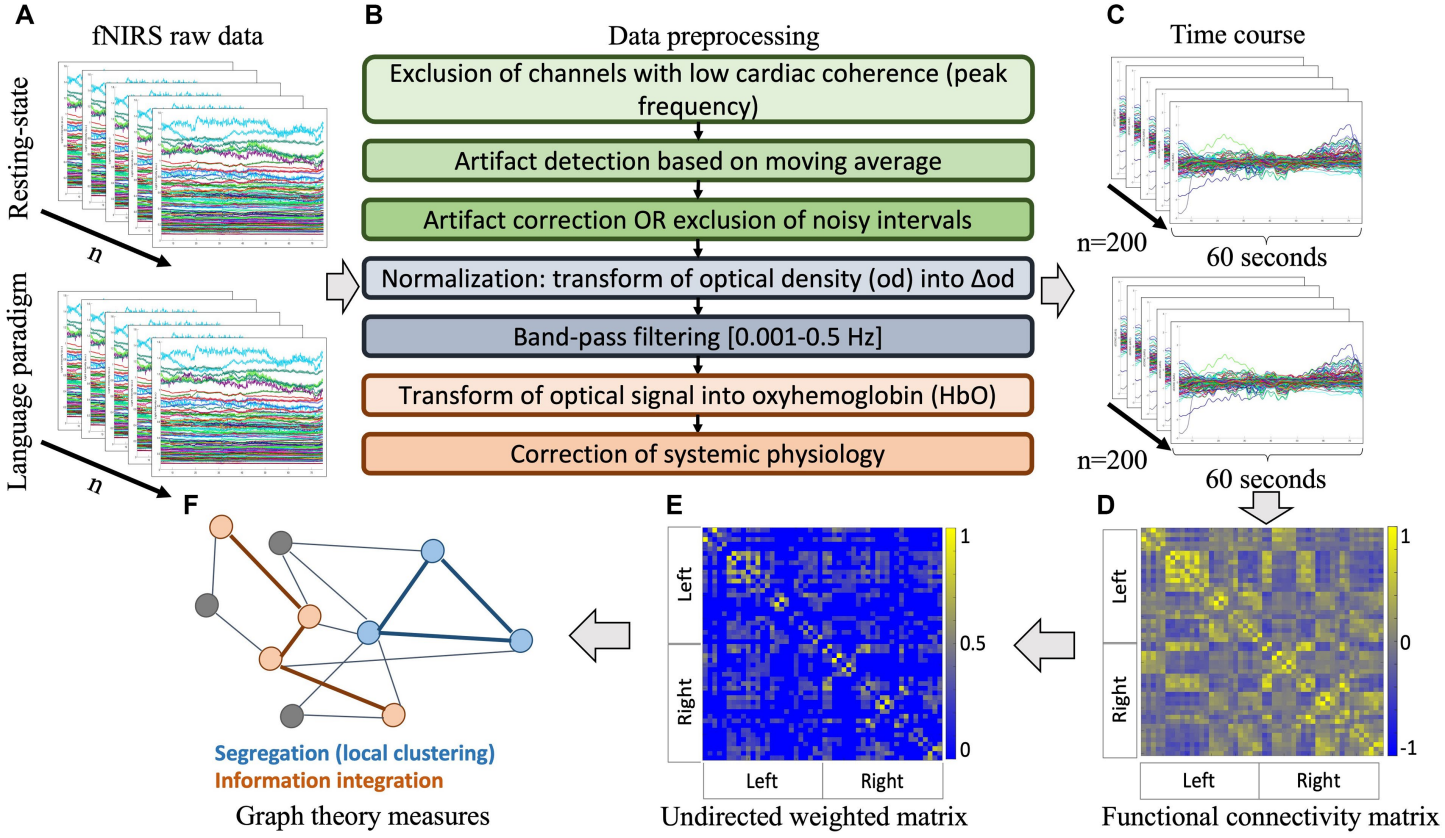
Processing pipeline for the fNIRS signal. **(A)** Individual raw data of the resting-state and receptive language task; **(B)** preprocessing steps applied to ensure signal quality and transform raw optical density into relative oxy- and deoxy-hemoglobin concentration changes; **(C)** extraction of random samples of 60 s; **(D)** computation of cross-correlations between all channels of each sample to produce averaged individual functional connectivity matrices on oxyhemoglobin signal ( $A_{n}$ ); **(E)** thresholding of individual matrices over a sparsity range to produce undirected weighted graphs; **(F)** derivation of graph theoretical metrics to describe network properties. fNIRS: functional near-infrared spectroscopy, HbO: oxyhemoglobin.

Raw fNIRS data was imported into the LIONirs toolbox (Tremblay et al., 2022), which is embedded in SPM12 (Statistical Parametric Mapping) and operates in MATLAB (The MathWorks, Inc., 2019b, Natick, MA, United States). Signal coherence among channels for the peak frequency commonly related to the cardiac beat (0.8–2.3 Hz) allowed to exclude channels with poor optode-to-scalp coupling (Yücel et al., 2021; Fourdain et al., 2023). Intervals with aberrant signal variation based on moving average criteria were identified using an automatic algorithm. An artifact was identified if (a) changes in light intensity within a time interval of 6 s exceeded three standard deviations, (b) the noise lasted for more than 3 s, and (c) at least 5% of channels were affected. Temporally correlated ( r>0.8 ) channels within the same interval were considered as artifacts and intervals less than 2 s apart were considered as one event. A visual inspection was also performed, and minor manual adjustments were applied. Subsequently, parallel factor analysis (PARAFAC), i.e., a multidimensional decomposition method (Harshman, 1970; Bro, 1997) which has recently been validated for fNIRS data (Harshman, 1970; Bro, 1997; Hüsser et al., 2022), was used to correct the signal during relatively isolated artifact intervals. Noisy periods that lasted for a relatively long interval and where no clear signature could be extracted were excluded. Optical density of the signal between uncorrected noisy intervals, was transformed into delta optical density to normalize the signal. A fourth-order zero-phase Butterworth band-pass filter with cut-off frequencies 0.001–0.5 was applied to reduce confounds of systemic physiology related to respiratory or cardiac pulsation, while keeping the low frequencies presumably related to the hemodynamic response (Pinti et al., 2019; Santosa et al., 2020). Optical density was then transformed into relative oxy−/deoxyhemoglobin (HbO/HbR) concentration changes using the modified Beer–Lambert law (Kocsis et al., 2006) with age-appropriate differential pathlength factors (DPF; Scholkmann and Wolf, 2013). The averaged signal of all channels was regressed from every channel, a procedure which has been shown to successfully remove remaining physiologic confounds appearing in frequency bands that are in the range of the hemodynamic signal and thus hard to filter (Saager and Berger, 2008). See Figure 2 for an overview of the fNIRS preprocessing pipeline.

### 2.4. Functional connectivity analysis

FC and network analyses were conducted on HbO matrices only, which is considered a sensitive indicator of regional oxygenation differences in response to cerebral activation (Homae et al., 2007) and has a good concordance with the fMRI BOLD signal (Sato et al., 2013; Tung et al., 2021). Circular bootstrapping was used to extract 200 random 60-s samples from each data set (resting-state and task) of each participant (Bellec et al., 2010). For each valid sample, we computed cross-correlations, i.e., pairwise Pearson’s correlations ( ρ ) between the time course of all measurement channels ( $y_{i},y_{j}=1,\ldots 50$ ). The symmetric matrix of each subject ( $A_{n}$ ) included the averaged $\rho_{ij}$ of all possible pairs (*k* = 1,225) over all samples. Correlation coefficients underwent Fisher’s transformation to obtain normally distributed values ( $z_{ij}$ ). Developmental effects were removed by estimating and regressing the contribution of age from $A_{n}$ with a general linear model (GLM): $A_{n}=\beta_{0}+\beta_{1}\left(\mathrm{age}\right)+\epsilon$ , resulting in $B_{n}$ . The hemodynamic signal related to receptive language processing was isolated by subtracting the FC matrix computed using the resting-state data from the FC matrix computed using data gathered during the story listening task ( $B_{n\left(\mathrm{task}\right)}-B_{n\left(\mathrm{resting}-\mathrm{state}\right)}$ ). The subtraction allowed to control for inter-ictal epileptic or default mode network activity and yielded in a residual matrix ( $R_{n}$ ) specific to language comprehension.

The brain networks were characterized with common graph theoretical measures of local and global processing efficiency. Segregation is the network’s tendency to build local clusters and refers to local information processing. It is measured with: (1) the clustering coefficient ( γ ), which is the level a node’s neighbors are directly connected to each other and describes the clustering density around each node; and (2) the local efficiency (E_local_) index, which considers direct and indirect paths to describe a node’s integration among its neighbors (Watts and Strogatz, 1998; Latora and Marchiori, 2001, 2003; Newman, 2003; Achard and Bullmore, 2007; Fornito et al., 2016; Sporns, 2018). Integration describes a network’s ability to efficiently integrate information from distinct parts and refers to global information processing. It is commonly assessed using: (1) the characteristic’s path length (CPL, λ ), which quantifies the averaged shortest path between all possible nodes - a lower λ implies more rapid and efficient information processing; and (2) the global efficiency (E_global_) index, which also refers to the network’s global information exchange and is the reciprocal of the λ ( $\frac{1}{\lambda }$ ) (Latora and Marchiori, 2001; Fornito et al., 2016). Additionally, the small-world index ( σ ), which is a network characteristic that considers the normalized balance of a network’s segregation and integration σ=γ/λ (Watts and Strogatz, 1998), was considered. A network with small-world properties is characterized by a good balance of global and local processing efficiency, which is inherent for a healthy brain network architecture since early infancy and throughout adulthood (Fransson et al., 2011; Fornito et al., 2016; Liao et al., 2017).

Network metrics ( γ , E_local_, λ , E_global_, σ ) were computed with in-house Matlab scripts incorporating functions from The Brain Connectivity Toolbox (Rubinov and Sporns, 2010). In the graph theoretical framework, each measurement channel ( i,j ) of the fNIRS data represents a node and every correlation pair ( $\rho_{ij}$ ) is an edge. Individual connectivity matrices ( $B_{n\left(\mathrm{task}\right)}$ ) computed from the language task signal only that were corrected for developmental effects, were transformed into undirected weighted graphs ( $W_{n}$ ) using a range of absolute correlation thresholds ( τ ). The ideal range is commonly achieved when a fully connected network, that is each node is at least connected with one other node (degree centrality [*K*]≥ 1), is accompanied by a small-world topology ( γ>1,λ>1,σ>1 or in small networks when γ > γ _rand_) (Montoya and Solé, 2002; Bassett et al., 2008; Rubinov and Sporns, 2010). These criteria were most appropriate for our set of data and have been tested in other studies (Provost et al., 2023; Roger et al., under review). In the current study, a satisfying mean minimal *K* of 1 was maintained from a threshold of 0.01–0.17 for the task matrices. Within that range, the average normalized γ and λ also fulfilled the criteria of 1. Small-world topology based on the criteria for small networks was also fulfilled for this range; the actual index was however only higher than 1 for thresholds above 0.12. Given the size of the cerebral network, i.e., <3,000 nodes, analyses were conducted for threshold ranges of $\tau_{1}=0.01$ , to $\tau_{m\left(\mathrm{task}\right)}=0.17\text{,}$ with Δτ=0.01. Correlations fulfilling the threshold criteria kept their absolute value ( $\left|\rho_{ij}\right|$ ). Correlations on the principal diagonal representing each channel’s correlation with itself and correlations below the threshold were set to 0. For every threshold, we extracted 100 random graphs, derived network metrics and calculated a group mean over all graphs and subjects ( $M_{\mathrm{rand}}$ ). We then divided individual metrics, derived from the real graph, by $M_{\mathrm{rand}}$ resulting in normalized metrics. Finally, we calculated the area under the curve (AUC) for the normalized network metrics over the sparsity range, which provided a valuable alternative to analyzing metrics for each threshold individually (Zhang et al., 2011; Lei et al., 2015; Suo et al., 2015; Ding et al., 2017).

### 2.5. Statistics

The statistical analyses were conducted using the R statistical software and Matlab functions integrated in the LIONirs toolbox. Between group analysis (epilepsy vs. control group) allowed to: (1) detect potential differences regarding the sample’s demographic characteristics (age, sex and SES) and socio-affective wellbeing measure with the Beck-Y questionnaire; (2) ensure equal fNIRS signal quality (the number of good trials included for FC matrices); and (3) statistically compare graph network metrics as well as cognitive performance. Student’s *t*-tests, or the non-parametric Mann–Whitney-U test when normality was violated, were used for continuous variables and Fisher’s exact test were used to analyze categorical variables.

Due to the high number of connectivity links (*n* = 1,225), 2000 unpaired permutation tests based on student t-test statistic were computed to compare the residual FC matrices of the epilepsy group to the control group (Galán et al., 1997; Lage-Castellanos et al., 2010). Insufficient power precluded quantitative analyses of patient subgroups (FLE vs. TLE, left vs. right epilepsy). Therefore, the mean and standard deviation of the control group’s residual FC matrices were used to transform the patients’ residual FC matrices into *z*-scores. The *z*-scores of four different patient subgroups, i.e., FLE, TLE, left and right lateralized epilepsy, were averaged to qualitatively evaluate the impact of epilepsy lateralization and localization on organization of cerebral language networks.

Pearson’s correlation analysis between cognitive measures (estimated IQ, expressive and receptive language skills) and the AUC of the graph metrics were used to better understand the associations between network organization and the neuropsychological profile. Subsequently, linear regression models with the cognitive measures and the group factor (epilepsy and healthy control) as predictor variables, were tested to evaluate their relative explanation of the different network properties, i.e., the AUC of the network’s segregation ( γ , E_local_) of each hemisphere, the network’s integration ( λ , E_global_), and its small-world topology ( σ ). Post-hoc analyses were applied to disentangle the moderating effect of the group factor on the association between cognitive measures and the AUC of the different brain network. Explorative correlation analyses between clinical factors and the AUC of the graph metrics of epilepsy patients were computed to explore the impact of the type of epilepsy (frontal/temporal), epilepsy lateralization (left/right/bilateral), age of epilepsy onset, epilepsy duration and seizure control on the language brain network topology. Pearson’s correlation coefficients and Spearman rank correlations were used for continuous and categorical variables, respectively. Results are all interpreted with a critical alpha level of 0.05 and common effect size measures, i.e., Cohen’s *d*, partial eta squared ( $\eta_{p}^{2}$ ), and Varga and Delaney’s *A* (Cohen, 1988; Vargha and Delaney, 2000).

## 3. Results

All 49 participants completed the neuropsychological assessment and the resting-state fNIRS-EEG recording. However, fNIRS-EEG recording during the passive story listening task was not done or not completed in 9 participants (7 epilepsy patients and 2 healthy controls) due to participant discomfort, limited collaboration, or time restrictions. Since it was not possible to compute the residual matrix in those participants, they were excluded for all cerebral network analyses (Sections 3.2 and 3.4). The EEG of one healthy control suggested potential epileptiform abnormalities. Consequently, this participant was excluded from the study. The neurologist did not identify subclinical seizures in any epilepsy or control participant. Indicators of inter-ictal activity, such as spikes or slow waves, were identified in the resting-state signal of five patients and in the task signal of three patients. These events were short ( ≈ 0.002 s) and mostly limited to a single channel, thus minimally affecting the hemodynamic signal. The sample characteristics and neuropsychological results are reported in Sections 3.1 and 3.3.

### 3.1. Socio-demographic and clinical characteristics of the final sample

The final sample was composed of 13 children with epilepsy and 26 healthy controls (see Table 1 for socio-demographic and clinical characteristics). Comparisons between healthy controls and epilepsy patients regarding their socio-demographic characteristics revealed no significant differences for sex (Fisher’s exact test: *p* = 0.728), age (*t*(37) = 1.30, *p* = 0.203, *d* = 0.44, small effect), socio-economic status operationalized as the family income (W = 155.5, *p* = 0.793, *A* = 0.46, very small effect) nor the mean parental education (W = 202.0, *p* = 0.311, *A* = 0.60, small effect). The BECK-Youth Inventory (BYI-II) was only completed by participants aged ≥7 years, i.e., 10 epilepsy patients and 23 healthy controls. Both groups have a socio-affective well-being within the normative range, with no statistically significant differences between groups for any of the three dimensions of the BYI-II, i.e., self-concept (*t*(31) = 0.78, *p* = 0.439, *d* = 0.30, small effect), depression (*t*(31) = −0.93, *p* = 0.343, *d* = 0.37, small effect) and anxiety (*t*(31) = −2.00, *p* = 0.056, *d* = 0.75, medium effect). We thus considered both groups as being comparable and did not include any of the socio-demographic nor the socio-affective factors as covariables for primary statistical analysis. The etiology of epilepsy was unknown in eight patients, potentially structural (e.g., atrophy, cyst, sclerosis) in three, though one had incomplete signs of a hippocampal sclerosis, and an auto-immune disease was suspected in one. Etiology could not be verified for one. None of the patients presented a self-limited epilepsy syndrome (Specchio et al., 2022). Nine patients received a monotherapy, and four a combination of two ASMs. Carbamazepine and levetiracetam were the most common ASMs in this study group and anyone received treatment with topiramate, which is known to affect language functions (Lee et al., 2003; Szaflarski and Allendorfer, 2012). Supplementary Table S1 for details on individual patients.

### 3.2. Cerebral networks

On average, we recorded 12.2 (SD: ±2.8) and 12.3 (SD: ±2.1) minutes of resting-state and task data, respectively. After the bootstrapping of 60-s trials, we extracted an average of 93.2 (SD: ±59.5) and 121.6 (SD: ±59.1) valid resting-state and 67.2 (SD: ±51.9) and 95.3 (SD: ±54.2) task segments for the epilepsy and control groups, respectively. The number of valid segments for the resting-state (*t*(46) = 1.63, *p* = 0.109, *d* = 0.48, small effect) nor the language paradigm (*t*(37) = 1.54, *p* = 0.131, *d* = 53, medium effect) statistically differed between groups. When the comprehension of the story line presented during the receptive language task was assessed, epilepsy patients gave significantly fewer correct answers (40%) as compared to the healthy controls (90%, W = 181, *p* = 0.002, *A* = 0.54, very small effect).

**Figure 3:**
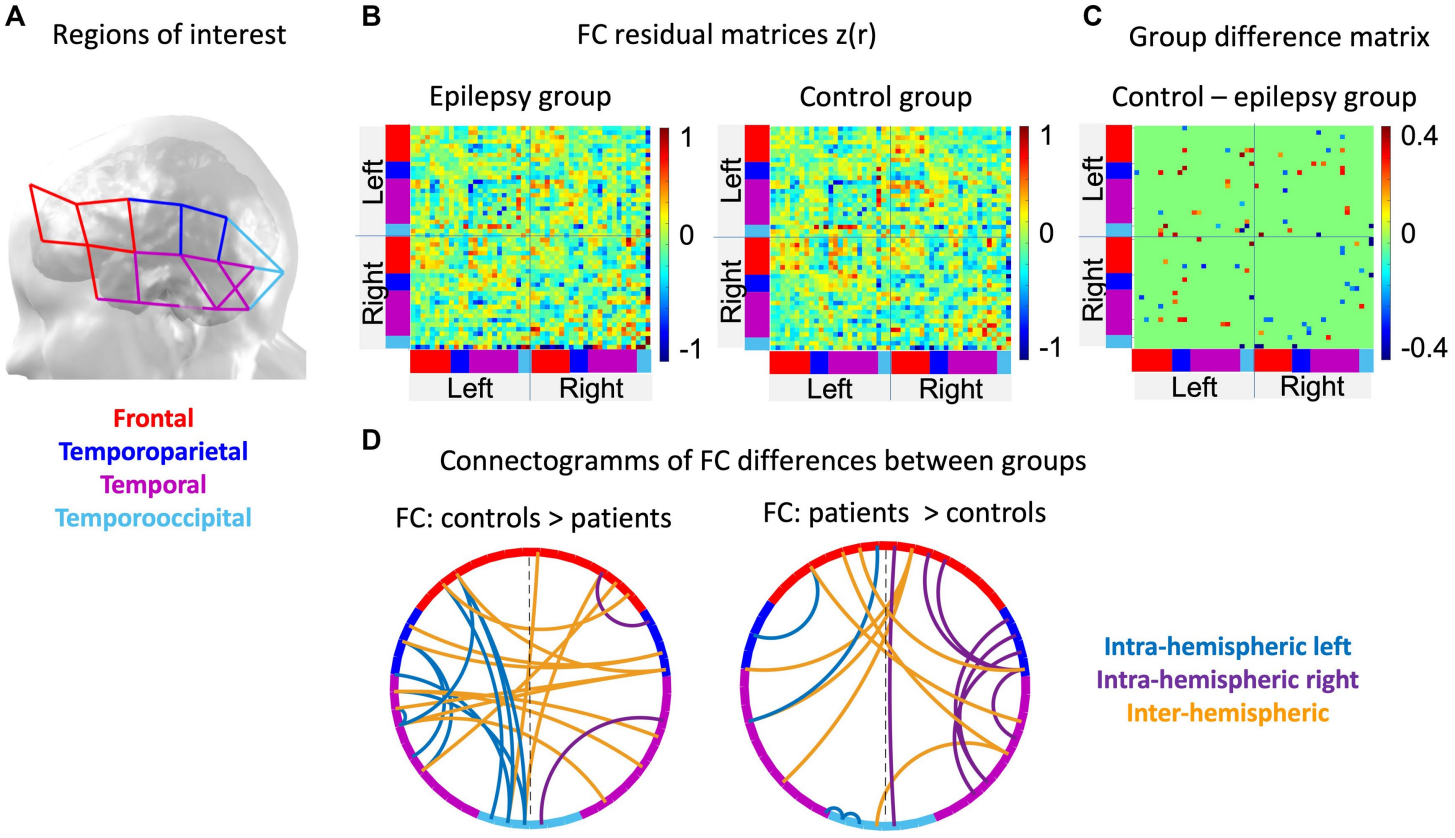
Results of functional brain networks involved in language processing: **(A)** Specifies how channels were regrouped into four regions of interest. The color codes of these regions are used to indicate regions in the connectivity matrices and the connectograms. **(B)** The averaged residual matrices (FC story – FC resting-state) are illustrated for the patient (left) and the control (right) group. **(C)** the group difference matrix shows the correlation pairs that differed significantly (*p* < 0.05) between both groups. **(D)** The connectograms separately show the significantly positive (left, patients < controls, Cohen’s *d* Min-Max: |0.74–2.81|, medium to large effect) and negative (right, patients > controls, Cohen’s *d* Min-Max: |0.74–2.07|, medium to large effect) differences of functional connections between groups, respectively.

**Figure 4:**
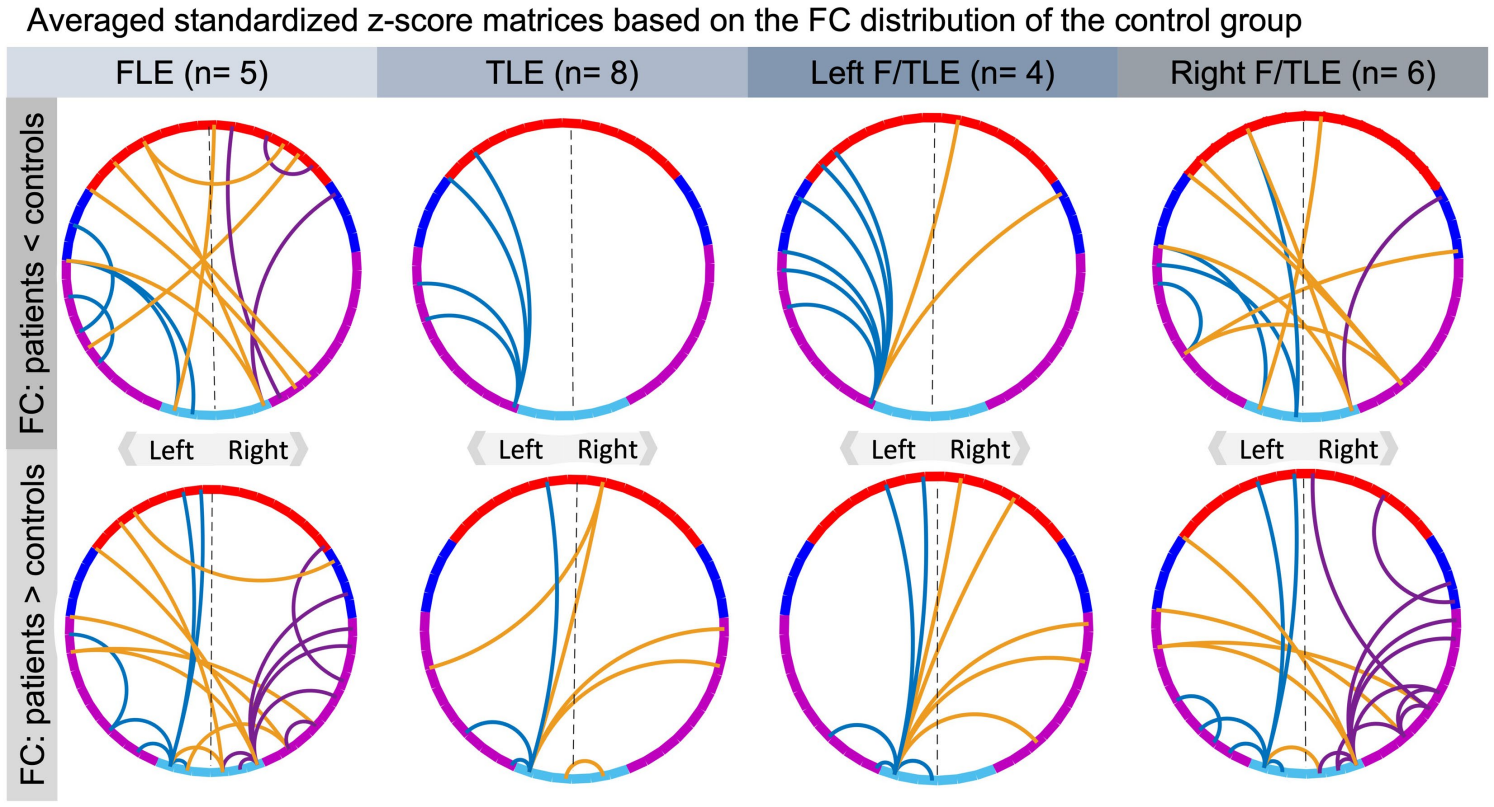
Qualitative results of averaged standardized residual matrices of patients’ subgroups. The upper part contains connections that are ≥2 SD lower in the epilepsy group than in the control group, while those in the bottom illustrate connections that are ≥2 SD higher in the epilepsy group compared to the control group. For all connectograms, the intra-hemispheric connections within the left and right hemisphere are displayed in blue and purple, respectively, and inter-hemispheric connections in orange. The color code of the regions of interest is specified in Figure 3A, where channels in red refer to frontal, blue to temporoparietal, purple to temporal and turquoise to temporoccipital areas of the brain.

Permutations were conducted at a channel-level, for visualization and interpretation purposes, channels were attributed to four regions of interest (ROI), i.e., frontal, temporoparietal, temporal and temporooccipital areas of both hemispheres (Figure 3A; Koessler et al., 2009; Nguyen et al., 2018). Averaged residual connectivity matrices ( R ) of epilepsy patients and healthy control children are illustrated in Figure 3B. Student’s *t*-test statistics after 2000 permutations revealed numerous significant differences (*p* < 0.05) between residual matrices of patients with epilepsy and healthy control children (Figure 3C). Group differences indicated that, compared to their healthy peers, children with FLE or TLE overall had decreased frontotemporal FC within the left hemisphere, increased frontotemporal FC within the right hemisphere, and mixed results regarding FC alterations of inter-hemispheric FC (*p* < 0.05, *d* |Min-Max|: 0.74–2.81, medium to large effect, see Figure 3D). Precisely, the epilepsy group predominantly had reduced FC between homologous regions (e.g., fronto-frontal connections) and a few stronger long-range inter-hemispheric connections (e.g., right frontal-left temporal) compared to the control group. The averaged *z*-score matrices for the four patient subgroups implied that children with FLE and right lateralized pathology, as compared to healthy controls, had the most different frontotemporal FC (see Figure 4). These patients had particularly increased FC of posterior temporal areas. Strikingly, children with left lateralized epilepsy had mainly left hemispheric and interhemispheric FC alterations, while their right hemisphere seemed almost unaffected and comparable to their healthy peers. The sample size prevented further statistical analyses between the subgroups.

**Figure 5:**
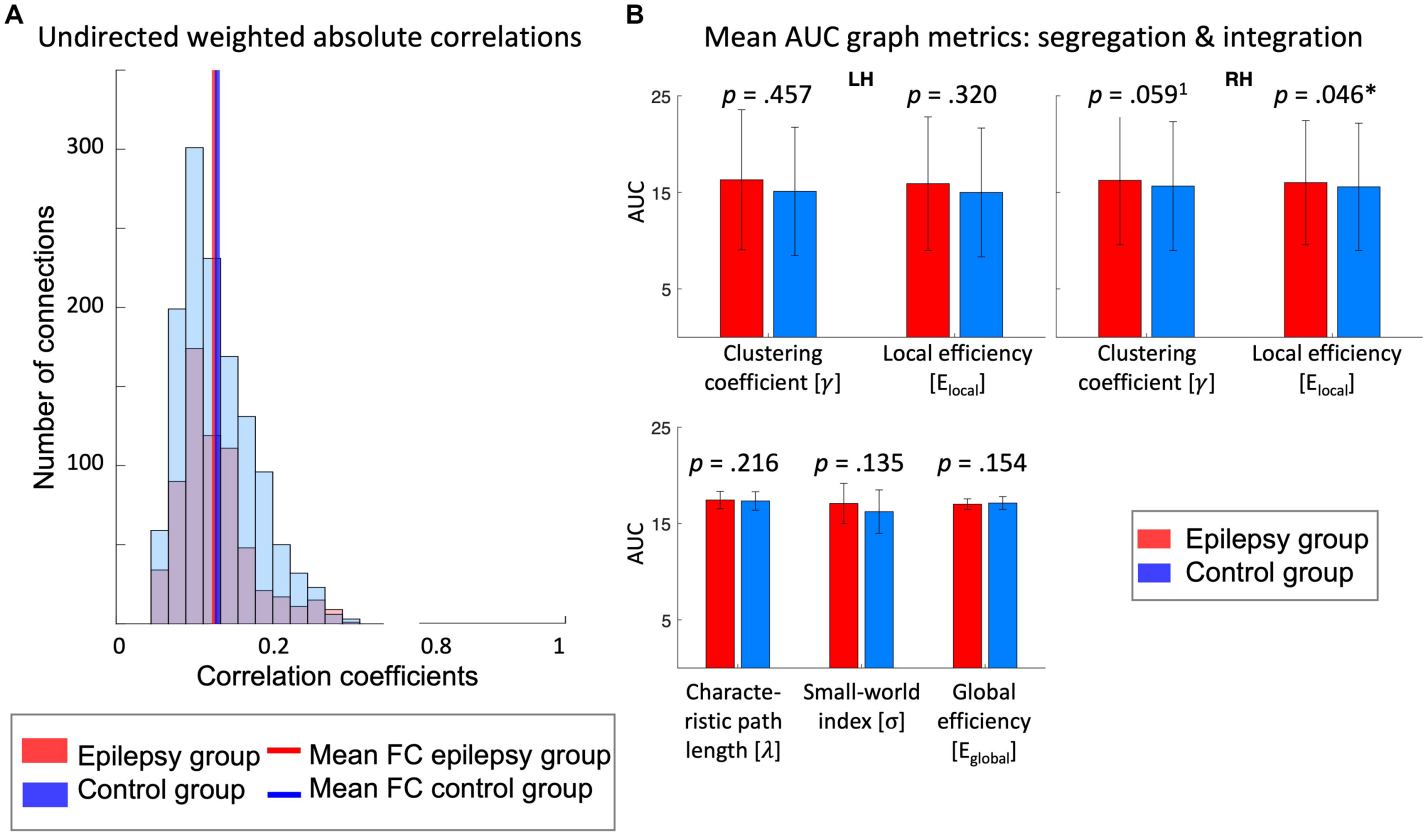
Graph analysis of functional brain networks involved in language processing: **(A)** The distribution of the functional connectivity strength and the mean functional connectivity as indicated by the undirected weighted absolute correlation coefficients across all channels of the receptive language task for the epilepsy (red) and control (blue) group. The overlap between the distribution of both groups appears purple. **(B)** The mean AUC of the local and global graph metrics, respectively, over the sparsity range of 0.01–0.17. Local metrics are displayed for the left and right hemisphere individually. AUC, area under the curve; FC, functional connectivity; LH, left hemisphere; RH, right hemisphere. *Critical alpha: *p = 0*.05, ^1^statistical tendency (0.05 < *p* < 0.08).

Connectivity strengths across all channels showed overall a similar distribution of FC. Averaged FC in the epilepsy and control group was 0.11 and 0.12, respectively (see Figure 5A). Weighted graph networks of children with FLE or TLE and healthy controls revealed a small-world topology ( γ>1,λ>1,γ > γ _rand_) for the language brain network over the sparsity range of $\tau_{1}=0.01$ , to $\tau_{m\left(\mathrm{task}\right)}=0.17$ . Figure 5B displays the averaged AUC for each graph network metrics. We found a significant group difference for the AUC of E_local_ in the right hemisphere (*t(37) =* −2.07, *p* = 0.046, *d* = −0.70, medium effect) and a relevant but not significant difference between groups for the clustering coefficient for the same hemisphere (*t*(37) = −2.07, *p* = 0.046, *d* = −0.66, medium effect), but no differences between patients and controls for any of the other network metrics (*p* > 0.05).

Explorative correlation analysis between some clinical factors and the AUC of the graph metrics of epilepsy patients revealed that overall epilepsy type showed the highest correlation across the AUC of all network metrics (Spearman *r* = |0.58–0.69|), followed by seizure control (Spearman *r* = |0.32–0.54|). Age of epilepsy onset (Pearson *r* = |0.20–0.33|) had small to moderate correlations with network metrics. Lateralization (Spearman *r* = |0.05–0.28|) and epilepsy duration (Pearson *r* = |0.00–0.10|) did not show any strong associations with the AUC of network metrics (see Table 2).

**Table 2:** Correlation analysis to assess the association of clinical factors and metrics of brain network organization.

	LH	RH	LH	RH	Characteristic path length	Global efficiency	Small-world index
	Clustering coefficient		Local efficiency				
Epilepsy type [FLE or TLE]^1^	−0.65	−0.58	−0.60	−0.58	0.69	−0.66	−0.66
Epilepsy lateralization [left, right, bilateral]^1^	−0.10	0.13	−0.05	0.18	−0.25	0.28	0.09
Age of epilepsy onset^2^	−0.20	−0.30	−0.24	−0.27	0.33	−0.30	−0.29
Epilepsy duration^2^	0.10	0.07	0.00	−0.09	−0.05	−0.01	0.07
Seizure control [yes, no]^1^	0.49	0.42	0.54	0.41	−0.36	0.32	0.47

^1^Spearman rank correlation, ^2^Pearson correlation coefficient, light grey, light blue/pale orange and dark blue/orange indicate small (*r* = |0–0.29|), medium (*r* = |0.30–0.49|), and large (*r* = |0.50–1.0|) correlations, respectively. Orange and blue stand for positive and negative correlations, respectively. LH, left hemisphere; RH, right hemisphere.

### 3.3. Cognitive profile

**Figure 6:**
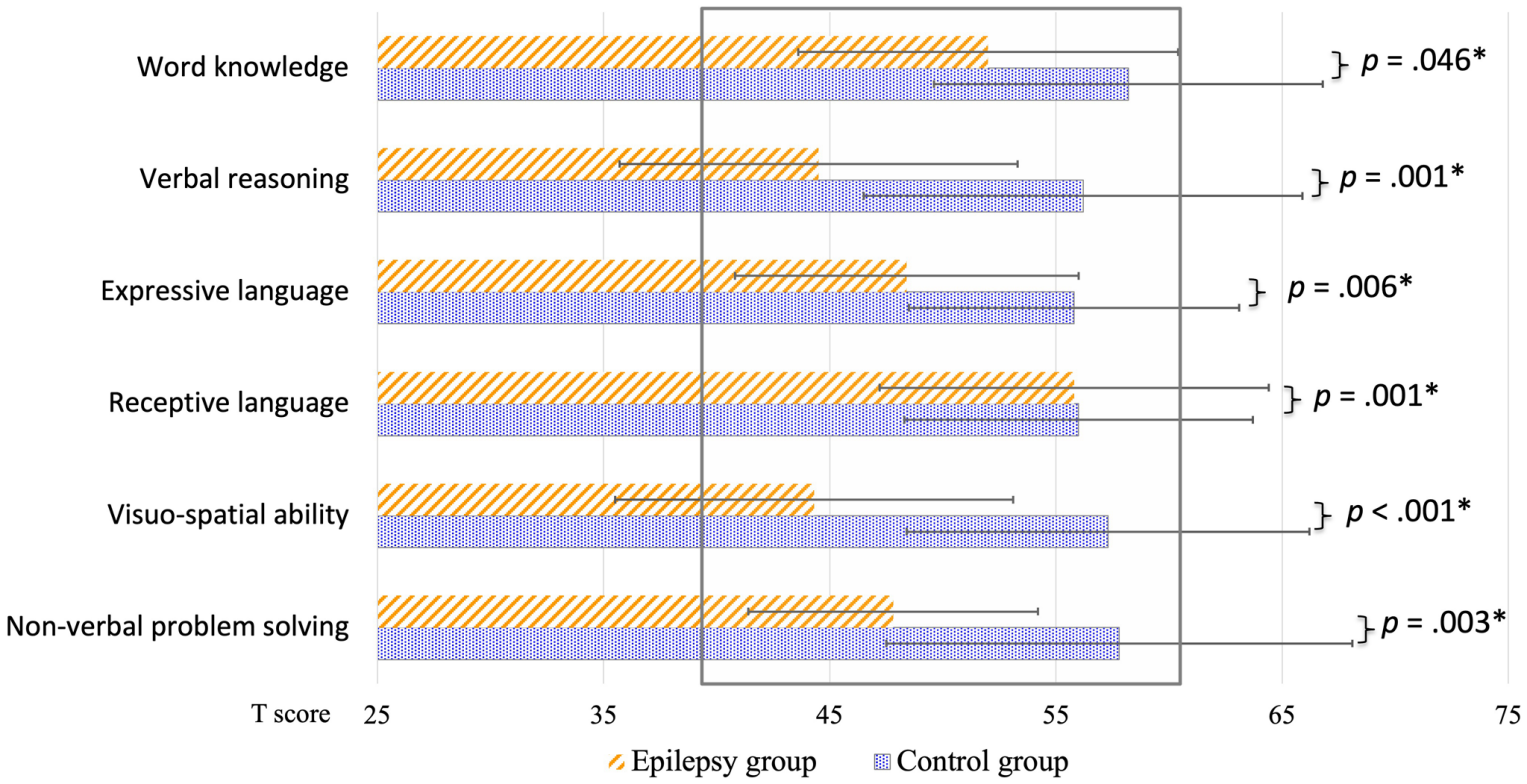
Group comparisons of cognitive performance: children with frontal or temporal lobe epilepsy (orange stripes) and healthy control children (blue dotted) are illustrated separately. Results are expressed as t-scores. The normative range is indicated with the grey rectangle (M ± SD = 50 ± 10). All comparisons had a medium to large effect size (*d* ≥ 0.7). *Critical alpha: *p* = 0.05.

Results of the neuropsychological evaluation revealed that, compared to children of the control group, children of the patients group had a significantly lower estimated global IQ (*t*(35) = 5.43, *p* < 0.001, *d* = 1.91, large effect, missing *n_epi/ctrl_*: 1/1), reduced overall verbal capacities (verbal comprehension index of the Wechsler intelligence scale, VCI: *t*(35) = 3.37, *p* = 0.002, *d* = 1.19, large effect, missing *n_epi/ctrl_*: 1/1), lower receptive (*t*(36) = 3.64, *p* < 0.001, *d* = 1.27, large effect, missing *n_epi_*: 1), and expressive language abilities (*t*(37) = 2.94, *p* = 0.006, *d* = 1.00, large effect) as well as decreased non-verbal skills (perceptual reasoning index of the Wechsler intelligence scale, PRI: *t*(37) = 4.78, *p* < 0.001, *d* = 1.62, large effect). Detailed results of the subtests are illustrated in Figure 6.

### 3.4. Association of brain network organization and the cognitive profile

Correlation coefficients revealed high correlations between the different cognitive measures (*r* > 0.70). Supplementary Table S2 provides the correlation coefficients between all variables. Due to multicollinearity between cognitive measures and because the estimated IQ had the most relevant and consistent association with network metrics across different metrics (*r* = |0.18–0.29|), we opted for a linear model that included the centered estimated IQ, the group factor and their interaction term to predict the AUC of the brain network metrics. Statistical results of this model are summarized in Table 3. 14.4% of the E_local_ variance in the left hemisphere (*F*(3,33) = 3.02, *p* = 0.044, *d* = 0.08, small effect) was explained by this model. 13.0% of the E_local_ variance in the right hemisphere (*F*(3,33) = 2.79, *p* = 0.056, *d* = 0.02, small effect) was explained by the included variables, which was however not statistically significant. We did not find any main effects of the group nor the estimated IQ, but there was a significant interaction effect between the group and estimated IQ for E_local_ of the left hemisphere (Beta = −1.46, *p* = 0.014, $\eta_{p}^{2}$ = 0.16, large effect) and an equally large but not significant effect for E_local_ of the right hemisphere (Beta = −1.29, *p* = 0.055, $\eta_{p}^{2}$ = 0.10, medium to large effect). The F-statistics of all the other metrics did not reveal a significant model fit.

**Table 3:** Regression analysis to assess the association of cognitive measures and metrics of brain network organization and to detect potential moderating effects of the group factor.

Dependent variables	Clustering coefficient		Local efficiency		Characteristic path length	Global efficiency	Small-world index
	LH	RH	LH	RH			
**Model fit**	AUC = ß0 + ß1(Estimated IQ) + ß2(Group) + ß3(Estimated IQ*Group)						
*F*(3,33)	1.47	1.95	**3.02**	**2.79**	1.66	1.11	2.19
Adjusted *R*^2^	0.04	0.07	**0.14**	**0.13**	0.05	0.01	0.09
*p*	0.242	0.140	0.044*	**0.056**^**1**^	0.195	0.360	0.108
**Estimated IQ**							
ß	−0.14	0.01	0.03	0.12	−0.04	0.03	0.02
*p*	0.745	0.984	0.915	0.714	0.816	0.745	0.972
**Group**							
ß	−0.49	0.53	−0.19	0.49	−0.06	0.09	0.10
*p*	0.489	0.492	0.676	0.360	0.819	0.489	0.890
Group*Estimated IQ							
ß	−1.41	−1.37	**−1.46**	**−1.29**	**0.59**	−0.20	**−1.80**
*p*	0.112	0.149	0.014*	**0.055**^**1**^	**0.080**^**1**^	0.223	**0.057**^**1**^

LH, left hemisphere; RH, right hemisphere. *Critical alpha: *p* = 0.05, ^1^statistical tendency (0.05 < *p* < 0.08). Significant effects and statistical tendencies are emphasized in bold.

**Figure 7:**
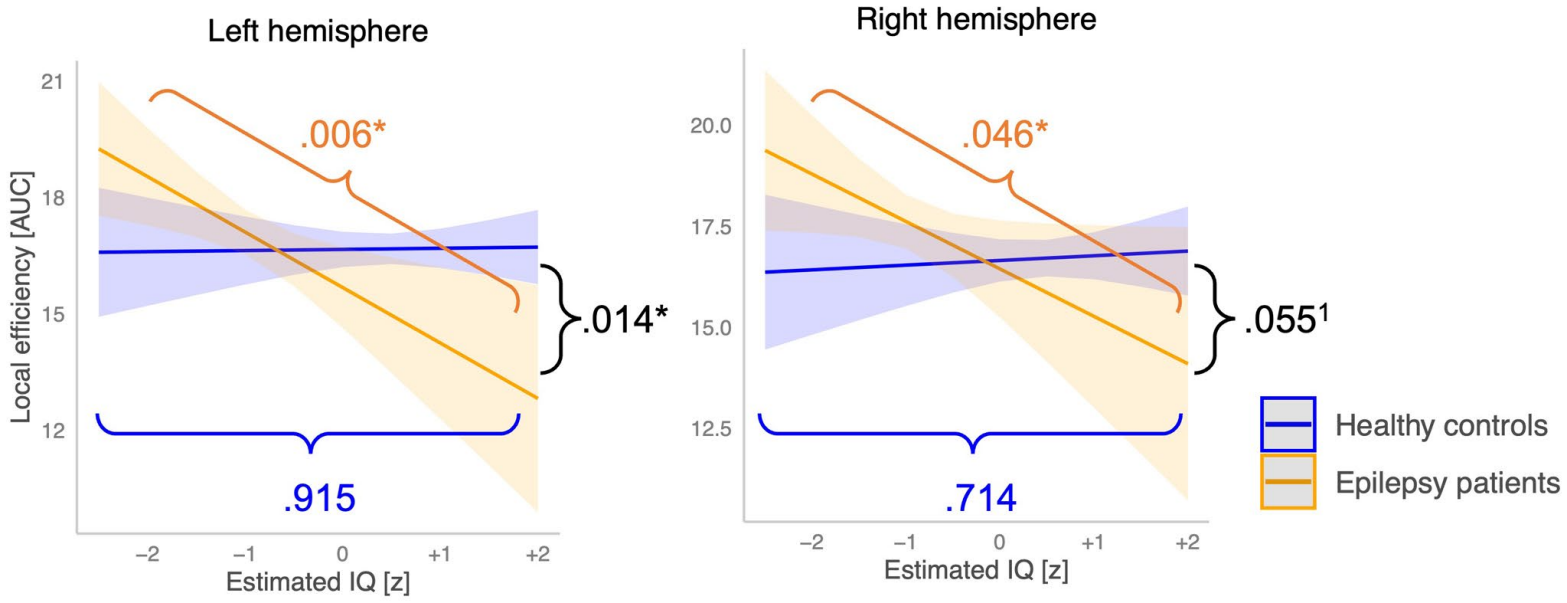
Moderation of the association between the estimated IQ and local network topology by the group factor. Orange lines indicate the between group difference in the slope, blue lines show the slope of the epilepsy group, and the green lines represent the slope of the control group. *Critical alpha *p* = 0.05, ^1^statistical tendency. (0.05 < *p* > 0.08).

*Post-hoc* simple slope analyses revealed that the association between the estimated IQ and E_local_ of the left hemisphere differed significantly between groups (*p* = 0.014) and at trend level for the E_local_ of the right hemisphere (*p* = 0.055). Precisely, an increase in the estimated IQ was associated with a significant decrease of E_local_ in the left (−1.43, *p* = 0.006) and the right hemispheres (1.17, *p* = 0.046) in the epilepsy group. In the control group, an increase of the estimated IQ did not relate to any changes of the E_local_ in the left (+0.03, *p = 0*.915) nor the right (+0.12, *p* = 0.714) hemispheres (see Figure 7).

## 4. Discussion

Developing language brain networks are vulnerable to neuropathologies, which may interrupt or disturb their normal development. At the same time, the developing brain benefits from great plasticity, offering a unique window for adaptive processes in response to neuropathologies (Smith, 2010). Differences in cerebral networks involving language processing have been reported in children with focal epilepsies compared to healthy controls (Hamberger and Cole, 2011; Sepeta et al., 2015; Slinger et al., 2022). However, specific patterns of language brain network alterations in pediatric patients with FLE and TLE are unknown and their associations with cognitive and language abilities are not fully understood. Therefore, it is not clear if the differences found in children with FLE or TLE compared to healthy controls represent positive adaptive reorganization or if they constitute pathological brain reorganization in response to epilepsy. The main objectives of this study were to characterize the cerebral language network organization and to study the associations of these networks’ characteristics with the cognitive and language profiles in children with FLE or TLE.

### 4.1. Cerebral networks

In the current study, the healthy control group presented typical cerebral language networks characterized by a joint recruitment of left temporoparietal, temporal and temporooccipital areas as well as of bilateral frontal and temporal areas (Skeide and Friederici, 2016; Hickok, 2022). Compared to healthy controls, children with FLE and TLE demonstrated weaker left hemispheric and inter-hemispheric FC as well as increased right frontotemporal connections. These group differences support the known incidence of atypical functional language networks in pediatric focal epilepsy (Berl et al., 2014; Baciu and Perrone-Bertolotti, 2015; Vannest et al., 2019; Marcelle et al., 2022). While the enhanced recruitment of right hemispheric frontotemporal regions is a frequent and well-established cerebral alteration of language processing in epilepsy patients, the impact on long-distance connections is less documented. In a recent fNIRS study in adult patients with frontotemporal epilepsy, Tung et al. (2021) investigated brain FC during a semantic and a phonemic verbal fluency task and found weaker inter-hemispheric FC in adult epilepsy patients compared to healthy controls. Our findings thus suggest that weaker inter-hemispheric FC between homologous frontotemporal regions in patients with FLE and TLE compared to controls is a characteristic of cerebral language network reorganization across ages and different language domains (Sepeta et al., 2015; Tung et al., 2021).

Graph theory analysis revealed that global network topology of language processing was comparable between both groups, but that local network properties, i.e., local processing efficiency, in the right hemisphere was increased in the patient group compared to the control group. This suggests that the altered FC patterns in our epilepsy group only marginally affected the network’s overall architecture and that the increase of right intra-hemispheric FC results in more segregation of right hemispheric frontotemporal regions. The reduction of FC within the left hemisphere does not however lead to significant changes in network architecture within this hemisphere. The typical developmental timeline of network architecture indicates that local network topology, i.e., network segregation, tends to decrease with age, while global network efficiency usually increases (Supekar et al., 2009; Gozdas et al., 2019). Hence, the above reported alteration could indicate a certain immaturity of brain language networks in children with FLE and TLE. Similarly, local network organization seems a common area of alterations in patients with epilepsy as the results of the meta-analysis by Slinger et al. (2022) underline. In contrast to our findings, they report however a lower clustering coefficient, suggesting a reduction of network segregation in adolescent and adult patients with focal epilepsy. Alterations of local network properties appear to be a key characteristic of brain network topology in patients with focal epilepsy such as FLE and TLE, and this both for large-scale brain networks as well as specific functional brain networks such as cerebral language networks. The direction of reorganization, i.e., whether fewer or more subnetworks are built, seems to depend on the patients’ age.

Exploratory analyses of cerebral language networks in patient subgroups revealed that, compared to healthy controls, children with right lateralized epilepsy had overall more altered FC in cerebral language networks than patients with left lateralized epilepsy. The latter mainly had reduced FC among ipsilateral frontotemporal regions, suggesting that their language networks demonstrate primarily local deviations from the FC of healthy controls. Although epilepsy lateralization reportedly causes distinct differences in language networks, the literature rather suggests that patients with left lateralized FLE and TLE, i.e., seizure onset in the dominant hemisphere for language processing, experience more important alterations in their language networks compared to those with right hemispheric lateralization (Hamberger and Cole, 2011; Rodríguez-Cruces et al., 2020; Tung et al., 2021). Our data and others show that right hemispheric epilepsy does however not seem to prevent reorganization of cerebral language networks, causing a distinct pattern of alteration (Smith, 2016; Tung et al., 2021). This is in line with the current understanding of the typical neurobiology of cerebral language networks that suggests that despite a left hemispheric dominance, bilateral frontotemporal areas are involved in language processing (Hickok and Poeppel, 2007; Hickok, 2009, 2022). Regarding the role of epilepsy type, our results indicate that FC in children with FLE was more strongly affected compared to those with TLE with much more differences in intra- and inter-hemispheric FC in FLE than TLE compared to healthy controls. A distinct pattern of FC for epilepsy type has recently also been identified in adult patients with FLE and TLE (Caciagli et al., 2023). Precisely, fMRI data acquired during two expressive language tasks indicate that patients with FLE compared to those with TLE demonstrate increased FC within left temporooccipital and bilateral occipital areas. In our data set, epilepsy type and to some extent seizure control, showed the strongest associations with graph metrics of network topology. Epilepsy type and lateralization seem relevant predictors for the organization of cerebral language networks, though more studies are necessary to better characterize the specific pattern of reorganization associated with each subgroup. In this context, it is also important to emphasize the heterogeneous seizure semiology. Precisely, cerebral language network organization may be affected differently when seizures are purely unilateral, or epileptic activity propagates to the other hemisphere or seizures commonly result in a secondary generalization. Future studies with larger samples size should consider more precise characteristics of seizure semiology.

### 4.2. Cognitive profile

The neuropsychological results indicated that children with FLE or TLE had an overall cognitive performance and receptive and expressive language capacities within the normative range, however significantly lower than their healthy peers. In our sample, FLE and TLE seem to have a general impact on the cognitive capacities rather than a specific effect on language abilities. This is in line with the current state of the literature on pediatric FLE or TLE, which shows that focal epilepsy is a network disorder and affects multiple cognitive domains (Garcia-Ramos et al., 2015; Kellermann et al., 2016; Hermann et al., 2017; Verche et al., 2018; Rodríguez-Cruces et al., 2020).

### 4.3. Relationships between brain network organization and cognitive profile

During the fNIRS-EEG recording, patients with epilepsy demonstrated reduced task performance at the passive story listening task, i.e., lower comprehension of the story line, compared to the control group. This suggests that the diffuse FC alterations and increased local processing efficiency in the right hemisphere measured during language processing seem to represent aberrant network organization. Specific regression analysis revealed however a more complex pattern. The estimated IQ alone did not predict any of the network’s characteristics. However, we found a moderation effect of the group factor (patients vs. controls) on the association between the estimated IQ and the network’s local processing efficiency in the left hemisphere and at a trend level in the right hemisphere. Precisely, the estimated IQ was negatively related to the level of local processing efficiency in the patient’s group, while no such relationship was identified for the control group. No moderation effect was found for the clustering coefficient nor any of the global network metrics. Although current findings from neurotypically developing children suggest that the direction of the association between intellectual capacities and graph theoretical measures differs between cerebral regions (Gozdas et al., 2019), network segregation generally decreases during childhood and adolescence (Supekar et al., 2009). The association of a lower network segregation with higher cognitive functioning may imply that compensatory mechanisms in the left hemisphere enabled a more mature and therefore more efficient cerebral language network.

### 4.4. Strengths and limitations

This study has several methodological strengths that can be highlighted here. First, epileptic activity can significantly affect hemodynamic fluctuations and mislead the interpretation of cerebral language processing. The simultaneous acquisition of the electrophysiological data (EEG) with the hemodynamic signal (fNIRS) thus allowed to monitor epileptic activity and ensure a good quality of the hemodynamic signal without contamination of seizure activity. In future studies, the joint multimodal analysis of EEG and fNIRS FC would certainly also offer rich information to characterize language brain networks in individuals with epilepsy. However, a task paradigm better adapted to both signals (slow fNIRS hemodynamic and fast EEG neuronal signals) and a greater number of EEG electrodes should be used. This nevertheless constitutes interesting paths for future research. Another methodological strength of this study is the subtraction of the resting-state FC matrices from the task FC matrices allowing to maximally isolate cerebral activation related to receptive language processing. Finally, a third strength is the use of a graph theoretical approach to characterize brain language networks in children with FLE and TLE, where such literature on this specific group of patients is still scarce.

This study also has several limitations that must be considered when interpreting its results. First, the heterogeneous clinical phenotype of epilepsy and the high number of comorbidities makes the recruitment of a homogeneous sample challenging and resulted in the current study in a relatively small sample. We carefully considered the sample size while performing statistical analysis and emphasize the value of effect sizes. The impact of few clinical factors (e.g., epilepsy type and lateralization, age at onset, duration of epilepsy, seizure frequency and control) on brain functional connectivity could only be addressed in an explorative manner and the sample size precluded their consideration for advanced analysis. Due to high heterogeneity, other clinical variables (e.g., etiologies, number, and type of ASM, etc.) could not be considered for analyses. It has however been shown that clinical factors correlate with the extent and type of cognitive difficulties (Jambaqué et al., 1993; Gallagher and Lassonde, 2005; Verche et al., 2018). Therefore, their role for cerebral language processing and the neuropsychological outcome should be specifically addressed in upcoming research with larger samples. This will allow to shed light on differences in brain network architecture of epilepsy patients and enhance the understanding of the role of clinical characteristics on the association between neuropsychological profiles and cerebral network organization. Finally, the current findings are deducted based on a story listening task. Therefore, whether they represent specific cerebral characteristics of receptive language, or rather a general effect of language processing must be analyzed in a study applying different language task paradigms. This would for instance allow us to compare neuronal response between receptive and expressive language processing or other distinct linguistic processes.

## 5. Conclusion

This is the first study that specifically focused on children with FLE and TLE, the most common types of focal epilepsies where epileptogenic networks are more likely to interfere with cerebral language processing. Another novelty is the use of graph theory measures to characterize network architecture of brain language networks and identify their relationship with cognitive measures. We found distinct patterns of FC for cerebral language processing in children with FLE or TLE compared to typically developing children. Precisely, children with FLE or TLE had decreased left frontotemporal connections, fewer inter-hemispheric connections between homologous regions and increased right intra-hemispheric frontotemporal connections compared to their healthy peers. This suggests an atypical cerebral representation of language networks, with greater involvement of the right hemisphere and less left and inter-hemispheric interactions in these children. The patients’ brain architecture was also characterized by higher local processing efficiency predominantly in the right hemisphere. These large-scale language network alterations in the epilepsy group were accompanied by a reduction of cognitive capacities both in verbal and non-verbal domains. In children with FLE or TLE, local efficiency in the left hemisphere was negatively associated with global intellectual functioning. Reduced network segregation, i.e., the tendency to build locally specialized clusters, especially in the left hemisphere, may thus be a specific aspect of cerebral reorganization allowed by early brain plasticity that overall seems to favor a better cognitive outcome. Future studies will allow to shed light on the associations between different measures of brain network alterations and the neuropsychological profile, and particularly address the complex impact of clinical factors on functional brain network organization, including factors such as epilepsy lateralization and localization, the age of epilepsy onset, epilepsy duration, seizure control or medication, on functional brain network organization. Understanding the developing brain’s adaptive capacities in response to neuropathologies and the long-term functional impact in the context of development has important fundamental and clinical implications. Our findings contribute to a better understanding of cerebral alterations in response to pediatric FLE or TLE and their functional impact and pave the way for further investigations. Epilepsy is a network disorder and understanding the broader picture of cerebral and cognitive alterations would enable the installation of more adequate interventions and individual support for these patients.

## Data availability statement

The raw data supporting the conclusions of this article will be made available by the authors, without undue reservation.

## Ethics statement

The studies involving humans were approved by research ethics board of the Sainte-Justine Mother and Child University Hospital. The studies were conducted in accordance with the local legislation and institutional requirements. Written informed consent for participation in this study was provided by the participants’ legal guardians/next of kin. Written informed consent was obtained from the minor(s)’ legal guardian/next of kin for the publication of any potentially identifiable images or data included in this article.

## Author contributions

AMH: Data curation, Formal analysis, Investigation, Methodology, Project administration, Visualization, Writing – original draft, Funding acquisition. PV: Investigation, Writing – review & editing. JT: Methodology, Software, Validation, Writing – review & editing. BO: Formal analysis, Writing – review & editing. AL: Writing – review & editing. PD: Writing – review & editing. PM: Writing – review & editing. ER: Writing – review & editing. KR: Investigation, Writing – review & editing. SF: Investigation, Writing – review & editing. SP: Investigation, Writing – review & editing. YM: Investigation, Writing – review & editing. DKN: Writing – review & editing. AG: Conceptualization, Funding acquisition, Resources, Supervision, Writing – review & editing.

## Abbreviations

FLE: frontal lobe epilepsy
TLE: temporal lobe epilepsy
FC: functional connectivity
fNIRS: functional near-infrared spectroscopy
EEG: electroencephalography
ASM: antiseizure medication
AUC: area under the curve
