# Supplementary material for: Brain language networks and cognitive outcomes in children with frontotemporal lobe epilepsy
Source: Front Hum Neurosci. 2023 Oct 27;17:1253529. doi: 10.3389/fnhum.2023.1253529 (PMC10641510; doi:10.3389/fnhum.2023.1253529)
Supplement: Supplementary file 1 [file Table_1.docx]

**Supplemental material**

# Table S1. Detailed clinical characteristics of patients with epilepsy

| Epilepsy localization | Etiology | Structural abnormalities | ASM at time of testing |
| --- | --- | --- | --- |
| Frontal left | Auto immune | Cerebral atrophy | Phenytoin, Clobazam |
| Temporal right | Unknown | None | Carbamazepine, Levetiracetam |
| Temporal left | Structural | Hippocampal sclerosis | Oxcarbazepine |
| Temporal left | Structural | Incomplete signs of hippocampal sclerosis | Carbamazepine |
| Frontal right | Unknown | None | Levetiracetam |
| Temporal right | Unknown | None | Carbamazepine |
| Frontal right | Unknown | None | Levetiracetam, Carbamazepine |
| Frontal bilateral | Unknown | None | Levetiracetam |
| Temporal bilateral | Unknown | Arachnoid cyst | Acid valproic |
| Temporal bilateral | Unknown | None | Levetiracetam |
| Temporal left | Unknown | None | Carbamazepine |
| Frontal right | N/A | N/A | N/A |
| Temporal right | Structural | White matter lesion, dysplasia | Lamotrigine |

ASM: antiseizure medication; N/A: not available.

# Table S2. Correlation analysis on the association of cognitive measures and metrics of brain network organization.

| Estimated IQ | | 1.00 |  |  |  |  |  |  |  |  |  |  |
| --- | --- | --- | --- | --- | --- | --- | --- | --- | --- | --- | --- | --- |
| Receptive language | | 0.76 | 1.00 |  |  |  |  |  |  |  |  |  |
| Expressive language | | 0.71 | 0.63 | 1.00 |  |  |  |  |  |  |  |  |
| fNIRS task performance^1^ | | 0.49 | 0.20 | 0.46 |  |  |  |  |  |  |  |  |
| Clustering coefficient | LH | -0.19 | -0.18 | -0.15 | -0.29 | 1.00 |  |  |  |  |  |  |
|  | RH | -0.28 | -0.09 | -0.20 | -0.39 | 0.77 | 1.00 |  |  |  |  |  |
| Local efficiency | LH | -0.23 | -0.19 | -0.22 | -0.36 | 0.93 | 0.75 | 1.00 |  |  |  |  |
|  | RH | -0.29 | -0.07 | -0.23 | -0.43 | 0.74 | 0.95 | 0.81 | 1.00 |  |  |  |
| Characteristic path length | | 0.21 | 0.04 | 0.09 | 0.29 | -0.53 | -0.52 | -0.63 | -0.61 | 1.00 |  |  |
| Global efficiency | | -0.18 | 0.02 | -0.07 | -0.24 | 0.25 | 0.25 | 0.44 | 0.43 | -0.91 | 1.00 |  |
| Small-world index | | -0.26 | -0.11 | -0.19 | -0.37 | 0.91 | 0.92 | 0.91 | 0.91 | -0.73 | 0.48 | 1.00 |
|  | | Estimated IQ | Receptive language | Expressive language | fNIRS task performance^1^ | LH | RH | LH | RH | Characteristic path length | Global efficiency | Small-world index |
|  |  |  |  |  |  | Clustering coefficient | | Local efficiency | |  |  |  |
|  | | **Cognitive measures** | | |  | **Network organization** | | | | | | |

Pearson correlation coefficient: *r*, light grey: *r* = |0-0.29], pale blue/orange: *r* = |0.30-0.49] medium, blue/orange: *r* = |0.50-1.0] large, orange: positive correlations, blue: negative correlations. LH: left hemisphere, RH: right hemisphere, fNIRS: functional near-infrared spectroscopy, ^1^estimated as percentage of correct responses regarding the comprehension of the storyline.
